# FHL-1 interacts with human RPE cells through the α5β1 integrin and confers protection against oxidative stress

**DOI:** 10.1038/s41598-021-93708-5

**Published:** 2021-07-08

**Authors:** Rawshan Choudhury, Nadhim Bayatti, Richard Scharff, Ewa Szula, Viranga Tilakaratna, Maja Søberg Udsen, Selina McHarg, Janet A. Askari, Martin J. Humphries, Paul N. Bishop, Simon J. Clark

**Affiliations:** 1 Division of Evolution and Genomic Sciences, School of Biological Sciences, Faculty of Biology, Medicine and Health, University of Manchester, Oxford, UK; 2 Panum Institute, Department of Immunology and Microbiology, University of Copenhagen, Copenhagen, Denmark; 3 Wellcome Centre for Cell-Matrix Research, Division of Cell Matrix Biology & Regenerative Medicine, School of Biological Sciences, Faculty of Biology, Medicine and Health, Manchester Academic Health Sciences Centre, University of Manchester, Oxford, UK; 4 Manchester Royal Eye Hospital, Manchester University NHS Foundation Trust, Manchester Academic Health Sciences Centre, Manchester, UK; 5 Lydia Becker Institute of Immunology and Inflammation, Faculty of Biology, Medicine and Health, University of Manchester, Oxford, UK; 6 Institute for Ophthalmic Research, Eberhard Karls University of Tübingen, Elfriede-Aulhorn-Straße 7, 72076 Tübingen, Germany; 7 University Eye Clinic, Department for Ophthalmology, University of Tübingen, Tübingen, Germany

**Keywords:** Extracellular matrix, Integrins, Complement cascade, Gene expression

## Abstract

Retinal pigment epithelial (RPE) cells that underlie the neurosensory retina are essential for the maintenance of photoreceptor cells and hence vision. Interactions between the RPE and their basement membrane, i.e. the inner layer of Bruch’s membrane, are essential for RPE cell health and function, but the signals induced by Bruch’s membrane engagement, and their contributions to RPE cell fate determination remain poorly defined. Here, we studied the functional role of the soluble complement regulator and component of Bruch’s membrane, Factor H-like protein 1 (FHL-1). Human primary RPE cells adhered to FHL-1 in a manner that was eliminated by either mutagenesis of the integrin-binding RGD motif in FHL-1 or by using competing antibodies directed against the α5 and β1 integrin subunits. These short-term experiments reveal an immediate protein-integrin interaction that were obtained from primary RPE cells and replicated using the hTERT-RPE1 cell line. Separate, longer term experiments utilising RNAseq analysis of hTERT-RPE1 cells bound to FHL-1, showed an increased expression of the heat-shock protein genes *HSPA6*, *CRYAB*, *HSPA1A* and *HSPA1B* when compared to cells bound to fibronectin (FN) or laminin (LA). Pathway analysis implicated changes in EIF2 signalling, the unfolded protein response, and mineralocorticoid receptor signalling as putative pathways. Subsequent cell survival assays using H_2_O_2_ to induce oxidative stress-induced cell death suggest hTERT-RPE1 cells had significantly greater protection when bound to FHL-1 or LA compared to plastic or FN. These data show a non-canonical role of FHL-1 in protecting RPE cells against oxidative stress and identifies a novel interaction that has implications for ocular diseases such as age-related macular degeneration.

## Introduction

**Figure 1:**
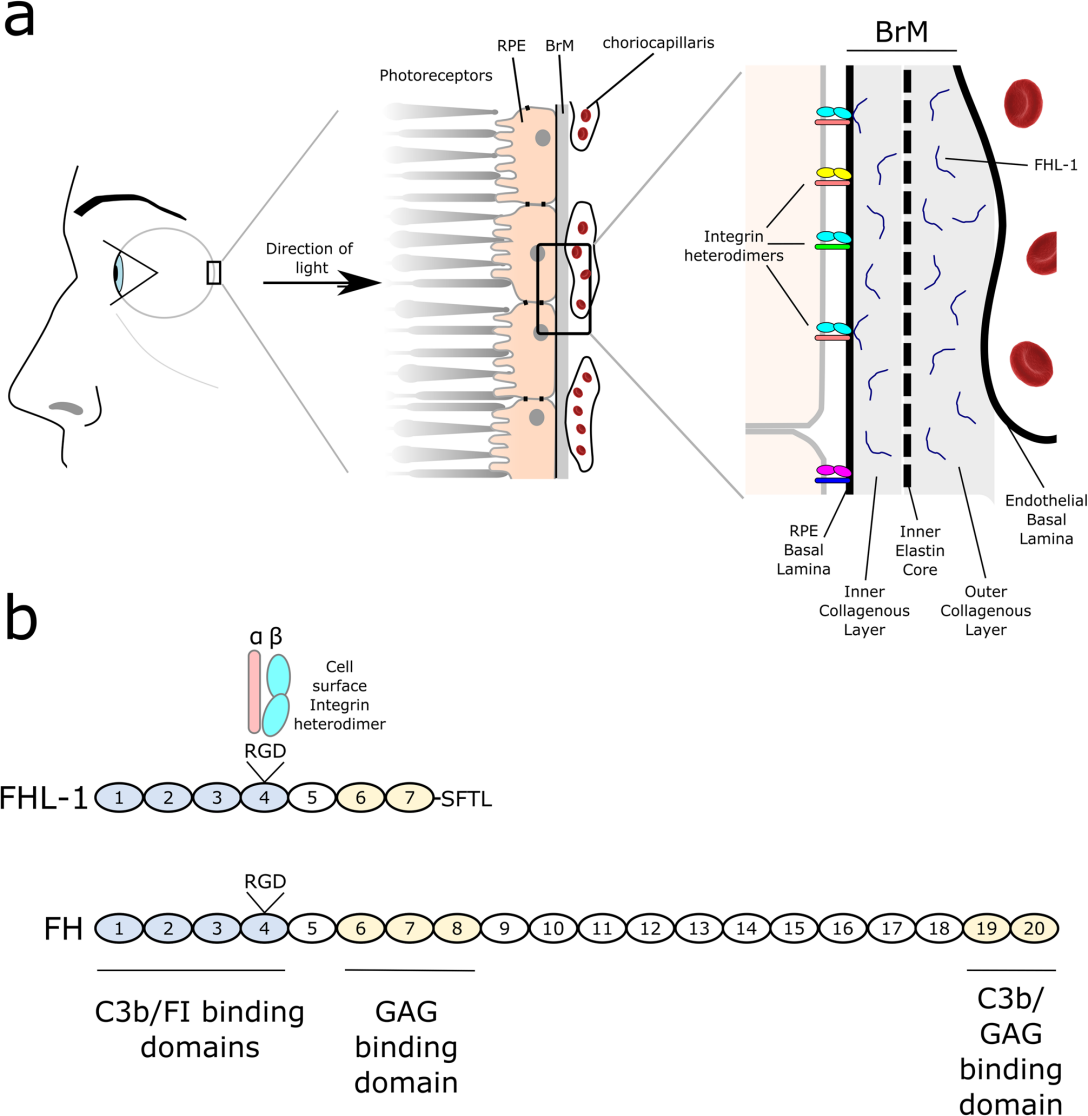
Schematic of RPE cell interactions with their underlying ECM. (**a**) The RPE forms a monolayer on Bruch’s membrane and plays a crucial role in maintaining photoreceptors. Bruch’s membrane separates the RPE from its blood supply, the choriocapillaris. RPE cells adhere to, and interact with, ligands within the basement membrane of Bruch’s membrane (BrM), including FN and LA, through various integrin heterodimer receptors expressed on their surface. Bruch’s membrane comprises five separate layers: the RPE basement membrane; the inner collagenous layer; an elastin core; the outer collagenous layer; and the endothelial basement membrane layer. The complement regulator FHL-1 is found anchored to heparan sulphate and dermatan sulphate glycosaminoglycan (GAG) chains found in collagenous and basement membrane layers of Bruch’s membrane. (**b**) Both FH and its truncated variant FHL-1 are comprised of complement control protein (CCP) domains: FH has twenty domains, while FHL-1 has seven CCPs identical to FH but with a unique C-terminal tail. Both proteins also share an RGD integrin-binding motif in CCP4.

The retinal pigment epithelium (RPE), a monolayer of cells in the retina, makes an essential contribution to the maintenance and support of the photoreceptor cells, and hence vision itself^1^. Disruption to the normal homeostasis of RPE cells is linked to a range of retinal degenerative diseases including age-related macular degeneration (AMD)^2^, the third leading cause of blindness in the world^3^. RPE cells have a high metabolic turnover and are exposed to extreme levels of light-induced oxidative stress^4^. Furthermore, these cells are among the most actively phagocytic cells in the body where they deal with the constant shedding of the outer segments of both rod and cone cells, which are dynamic structures that undergo constant renewal^5^. RPE cells also play fundamental roles in the transportation of nutrients from the underlying blood vasculature (termed the choriocapillaris, see Fig. 1a) to photoreceptors, and vice versa, such as the transportation of ions, water and metabolic end-products from the sub-retinal space to the blood.

RPE cells are separated from the choriocapillaris by an acellular barrier called Bruch’s membrane (Fig. 1a). This extracellular matrix (ECM) is comprised of five separate layers; the RPE basement membrane, inner collagenous layer, an elastin core, the outer collagenous layer, and the choriocapillaris basement membrane^6^. The structure and permeability of Bruch’s membrane is important for maintaining a healthy environment in the eye. Bruch’s membrane itself confers some selectivity as to what can, or cannot, pass through from the choroid to the retinal space^7^, leading to two immunologically semi-independent regions. Indeed, the breakdown of Bruch’s membrane barrier function allows the intrusion of blood vessels into the retinal space and is the site of lipid and debris accumulation that leads to the formation of drusen, the hallmark lesions of the early stages of AMD^8^.

The attachment of RPE cells to their underlying Bruch’s membrane is required to maintain their homeostasis, and changes to this ECM affects RPE cell gene transcription, translation and protein secretion^9^. Attachment of the RPE to the inner basement membrane of Bruch’s membrane is mediated at least in part by integrins (Fig. 1a). Integrins are heterodimeric proteins comprising various combinations of α and β subunits that determine ligand-binding specificity^10,11^ and control the morphology and fate of the host cell^12^. A variety of integrins are expressed by the RPE^13^, including the α5β1 integrin (*ITGA5:ITGB1*) which has been shown to mediate cell attachment, migration and proliferation^14^. The α5β1 integrin recognises the conserved binding motif Arg-Gly-Asp (RGD) that is present in various ECM ligands including fibronectin (FN)^15^.

Complement factor H-like protein 1 (FHL-1) is a truncated form of the complement inhibitory protein factor H (FH), which arises from alternative splicing of the *CFH* gene^16,17^. FHL-1, and to a lesser extent FH, have been identified within Bruch’s membrane and at the interface between Bruch’s membrane and the RPE^7,18^. Genetic variants in the *CFH* gene are associated with increased risk of AMD^19,20^ and this is thought to be due to decreased activity of FHL-1 and FH that results in increased complement activation in the ECM, leading to a local inflammatory response, the recruitment of circulating immune cells, formation of drusen, and ultimately RPE cell death^21–23^. However, recent studies have begun to identify non-canonical roles of FH and FHL-1 and the potential contribution of their dysregulation to AMD pathogenesis through inducing RPE mitochondrial dysfunction^24,25^ and lipid peroxidation^26^. Both FH and FHL-1 have an RGD motif on the surface of their fourth complement control protein (CCP) domain (see Fig. 1b) and it has previously been demonstrated that FHL-1 can confer cell attachment activity to human epithelial and fibroblast cell lines via this RGD motif^27^.

Herein, we investigate the interactions between primary human RPE cells, and the immortalised RPE cell line hTERT-RPE1, with immobilised FHL-1. We investigate the role of RPE cell integrins in interacting with FHL-1 and, by using RNAseq and cell survival assays, analyse the downstream consequences of the RPE cell/FHL-1 interaction compared to basement membrane components including laminin (LA) and FN. Subsequently, we elucidate a novel role for RPE/FHL-1 interactions in RPE cell modulation and their survival in response to oxidative stress.

## Results

### Primary human RPE cells interact with immobilised FHL-1

To investigate potential interactions between RPE cells and FHL-1, primary RPE cells were isolated from human donor cadaver eyes. Cells were examined for RPE marker expression by immunofluorescence, including ZO-1, RPE-65 and bestrophin-1 (Supplementary Fig. 1). Cell spreading assays are a commonly used methodology for investigating specific cell/ligand interactions^28^. These are performed within a short three-hour timeframe in order to avoid the deposition of endogenous ECM by the cells in question, thus masking the specific ligand-receptor interactions that are being investigated. Indeed, these cell spreading assays have been used previously with both RPE cells on various ECM^29,30^ and FHL-1 as a ligand for endothelial cells^27^, but not RPE cells and FHL-1 together.

**Figure 2:**
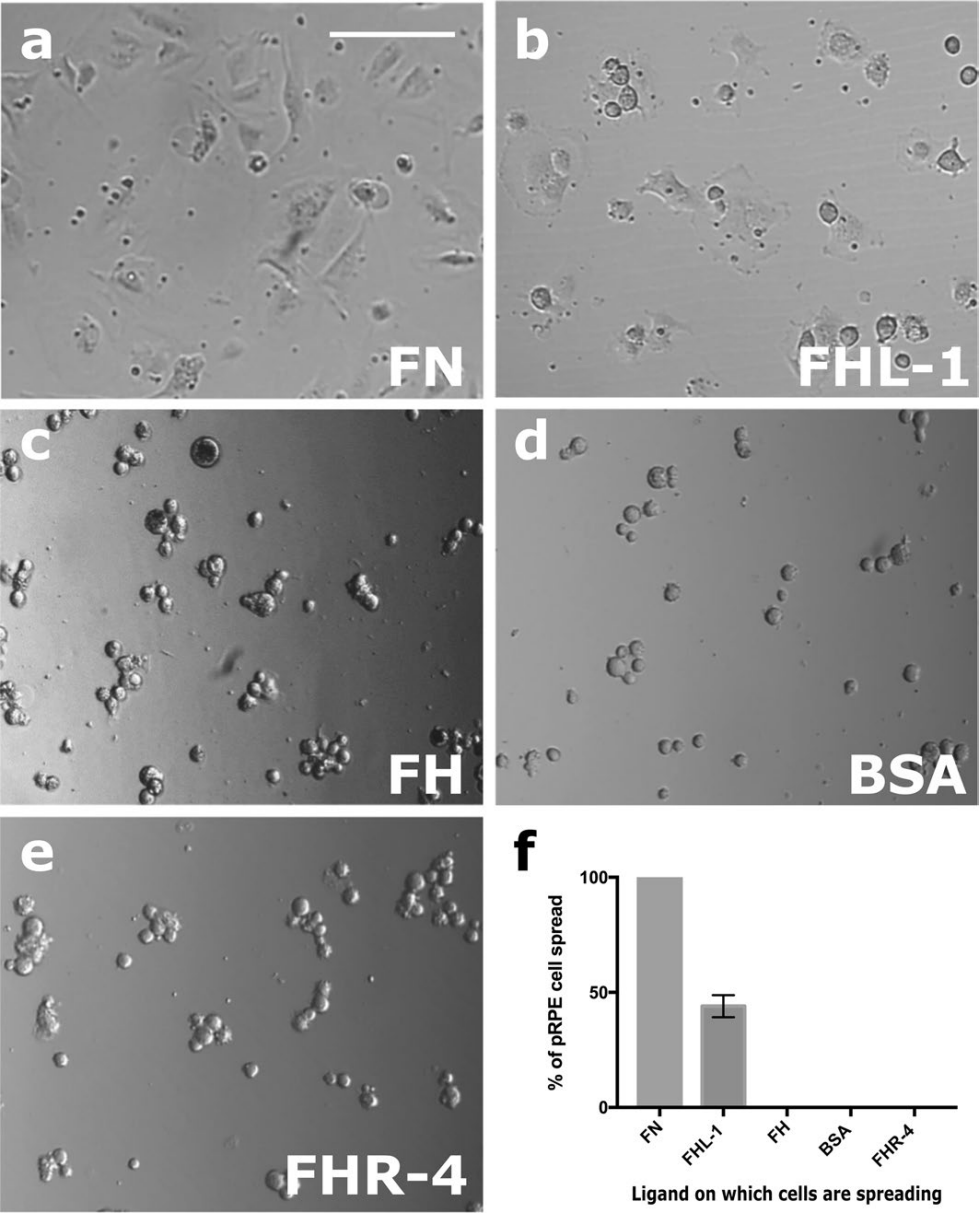
Primary RPE cells interact with immobilised FHL-1. Cultured primary RPE cells collected from human donor eyes were seeded at the same density (10,000 cells/well) in the wells of 96-well plates coated with: (**a**) FN; (**b**) FHL-1; (**c**) FH; (**d**) BSA; or (**e**) FHR-4. (**f**) the number of spread cells were counted in four different visual fields for each condition and each condition was measured in triplicate. The percentage of spread cells were calculated and compared to the positive control, fibronectin. Data are the average results of ten independent experiments and each performed in triplicate. Images in **a**-**e** are representative of 10 independent experiments. Incubation was done for 3 h at 37 °C. Scale bar represents 100 μm. Data in **f** represent n = 10 ± s.e.m.

Here, we used these assays to determine whether the primary RPE cells interact with FHL-1 immobilised onto a plastic surface. RPE cells were added to plates pre-treated with immobilised full-length FH or FHL-1 together with a FN positive control, and BSA and FHR-4 (a structurally related protein, but with no native RGD integrin binding motif) as negative controls. After three hours incubation, 40% primary RPE cell spreading was observed on FHL-1 compared to the FN control (Fig. 2). In contrast, primary RPE cells did not spread on immobilised full-length FH, despite it sharing an identical RGD binding site with FHL-1. Furthermore, no primary RPE cell spreading was visible on either plastic alone, BSA or FHR-4 (Fig. 2). Given that FH too has an RGD binding motif identical to FHL-1, the lack of RPE cell interaction seems perhaps surprising. To investigate further the lack of interaction with FH, cell spreading experiments were repeated in the presence of FH as a fluid phase competitor (see Supplementary Fig. 2). Additionally, FHL-1, FN and a recombinant protein comprising solely CCPs6-7 of FH were also used as fluid phase competitors. Fluid-phase FH inhibited the spreading of primary RPE cells on immobilised FHL-1, as well as fluid phase FHL-1 and FN (Supplementary Fig. 2). This suggests that the previously observed lack of RPE cell interaction with immobilised FH was due to the way the protein was absorbed onto plastic, and subsequent inaccessibility of its RGD domain, rather than an inherent lack of functional activity.

### RPE cell α5β1 integrin interacts with FHL-1 via its RGD site

**Figure 3:**
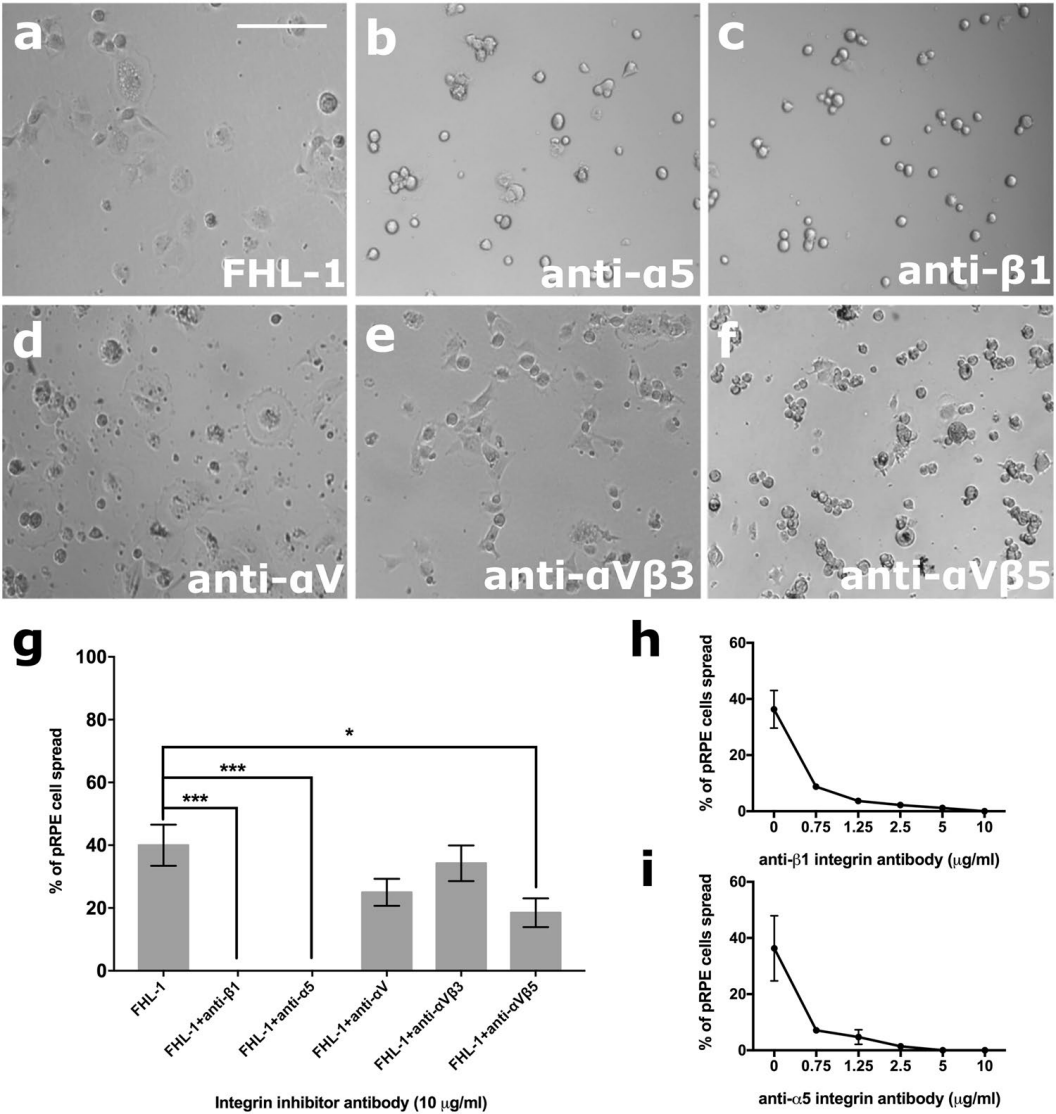
RPE cell interactions with FHL-1 are mediated through the α5β1 integrin. Cultured primary RPE cells (10,000 cells per well of a 96-well plate) were incubated on an FHL-1 matrix (**a**) in the presence of competing antibodies against specific integrin subunits (all 10 µg/ml), including: (**b**) anti-α5; (**c**) anti-β1; (**d**) anti-αV; (**e**) anti-αVβ3; and (**f**) anti-αVβ5. (**g**) The percentage of cell spreading on FHL-1 in the presence of each inhibiting antibody was calculated. The inhibitory effects of both anti-β1 and anti-α5 on primary RPE cell spreading on FHL-1 were shown to be dose dependent in (**h**) and (**i**) respectively. Data are the average results of three independent experiments and each performed in triplicate. Images in (**a**-**f**) are representative of 3 independent experiments. Data in (**g**) represent n = 3 ± s.e.m. Statistical analysis was performed by paired One-Way ANOVA with Tukey post hoc analysis, where *P < 0.05 and ***P < 0.001. Data in (**h**-**i**) are n = 3 ± s.e.m. Scale bar represents 100 μm.

Given the presence of an RGD motif in CCP4 of FHL-1 (Fig. 1b), we tested the role of integrins in FHL-1 binding. Competition assays were performed where primary RPE cells were bound to immobilised FHL-1 in the presence of inhibitory antibodies against integrins known to bind the RGD sequence including anti-α5 (mAb16), anti-β1 (mAb13), anti-αV (17e6), anti-αVβ3 and anti-αVβ5 (Fig. 3). The spreading of primary RPE cells on immobilised FHL-1 was completely abolished in the presence of either anti-α5 or anti-β1 antibodies in a dose-dependent manner (Fig. 3g-i). In addition, a significant reduction in RPE cell spreading was also achieved with the anti-αVβ5 antibody too (Fig. 3g), but given this integrins exclusive apical expression on RPE cells^31^, it is difficult at this time to hypothesise if this represents a true interaction that would be seen in vivo.

**Figure 4:**
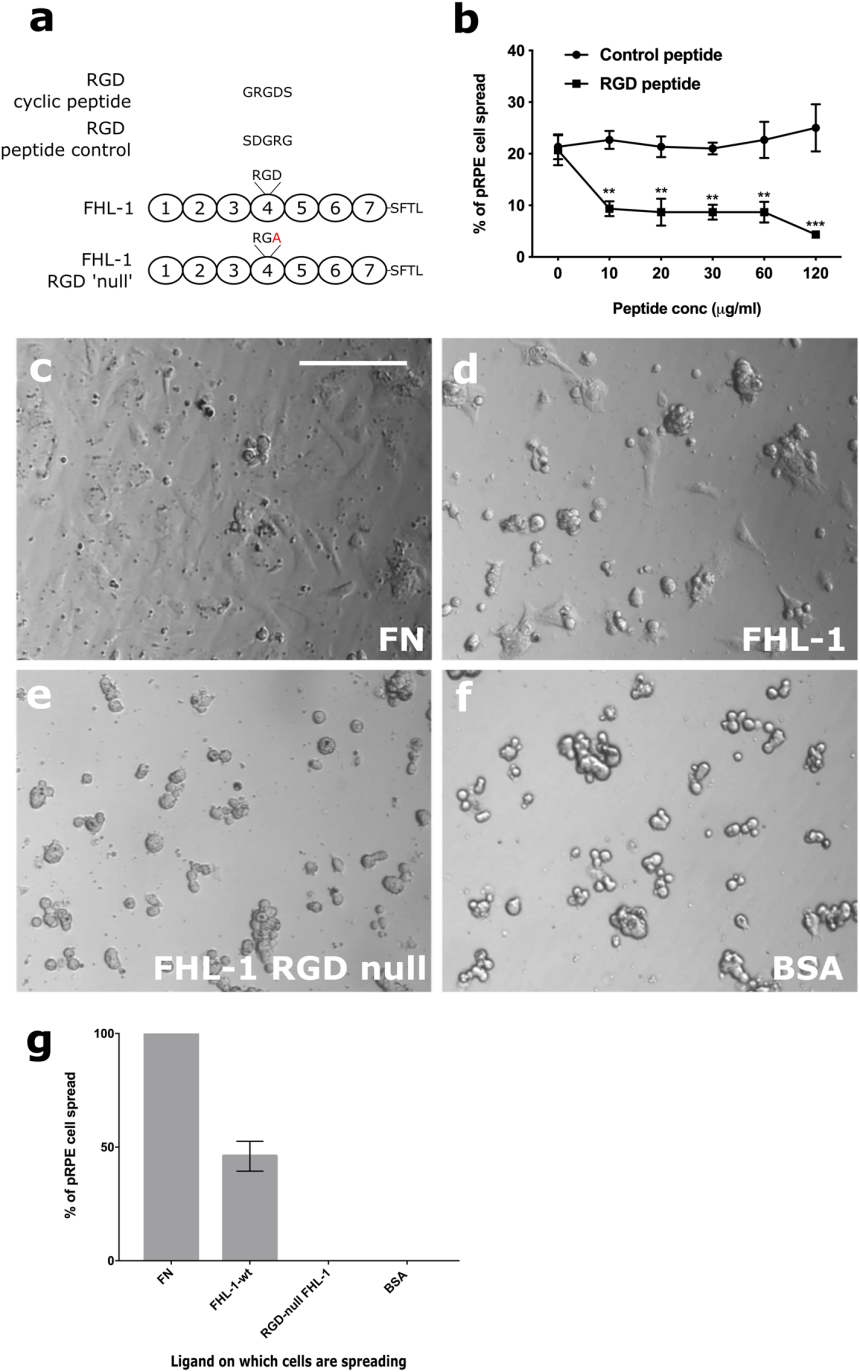
RPE cell α5β1 integrin recognises the RGD binding motif in FHL-1. (**a**) Schematic showing the design of both the RGD cyclic peptide and the scrambled peptide control, as well as the location of the aspartic acid mutation to an alanine residue for the creation of the RGD null FHL-1 protein. (**b**) The spreading of primary RPE cells to immobilised FHL-1 was inhibited with increasing concentrations of the cyclic RGD peptide: no inhibition was observed under the same conditions with the control scrambled peptide. Data comprises n = 3 ± s.e.m. Statistical analysis was performed using Student’s t test at each peptide conc., where **P < 0.01, and ***P < 0.001. The binding of the α5β1 integrin to the RGD binding domain in FHL-1 is tested by incubation of primary RPE cells with (**c**) fibronectin, (**d**) FHL-1, (**e**) FHL-1 RGD null, or (**f**) BSA. (**g**) Percentage of spread cells were calculated and compared to the positive control, fibronectin. Data are the average results of the three independent experiments and each performed in triplicate. Images in (**c**-**f**) are representative of 3 independent experiments. Data in (**g**) represent n = 3 ± s.e.m. Scale bar represents 100 μm.

In additional competition experiments, a cyclic GRGDS peptide decreased the percentage of spread RPE cells on FHL-1 compared to a reverse-sequence control peptide (SDGRG) (Fig. 4a-b). To confirm the role of the RGD domain in FHL-1 as an integrin binding site an FHL-1 RGD ‘null’ protein was made, where the aspartate residue in the RGD sequence was mutated to an alanine residue (making an RGA sequence, see Fig. 4a). The FHL-1-RGD null protein retained its functional co-factor activity for the complement factor I (FI) mediated breakdown of C3b into iC3b (see Supplementary Fig. 3), but lost all capacity to support primary RPE cell spreading (Fig. 4c-g). This lack of interaction was not due to the presence of any endotoxin contamination within the recombinant protein preparations as the measured endotoxin levels for FHL-1 and FHL-1 RGD null were 0.02 ng/ml and 0.03 ng/ml, respectively.

### The hTERT RPE-1 cell line mimics primary RPE cell behaviour

**Figure 5:**
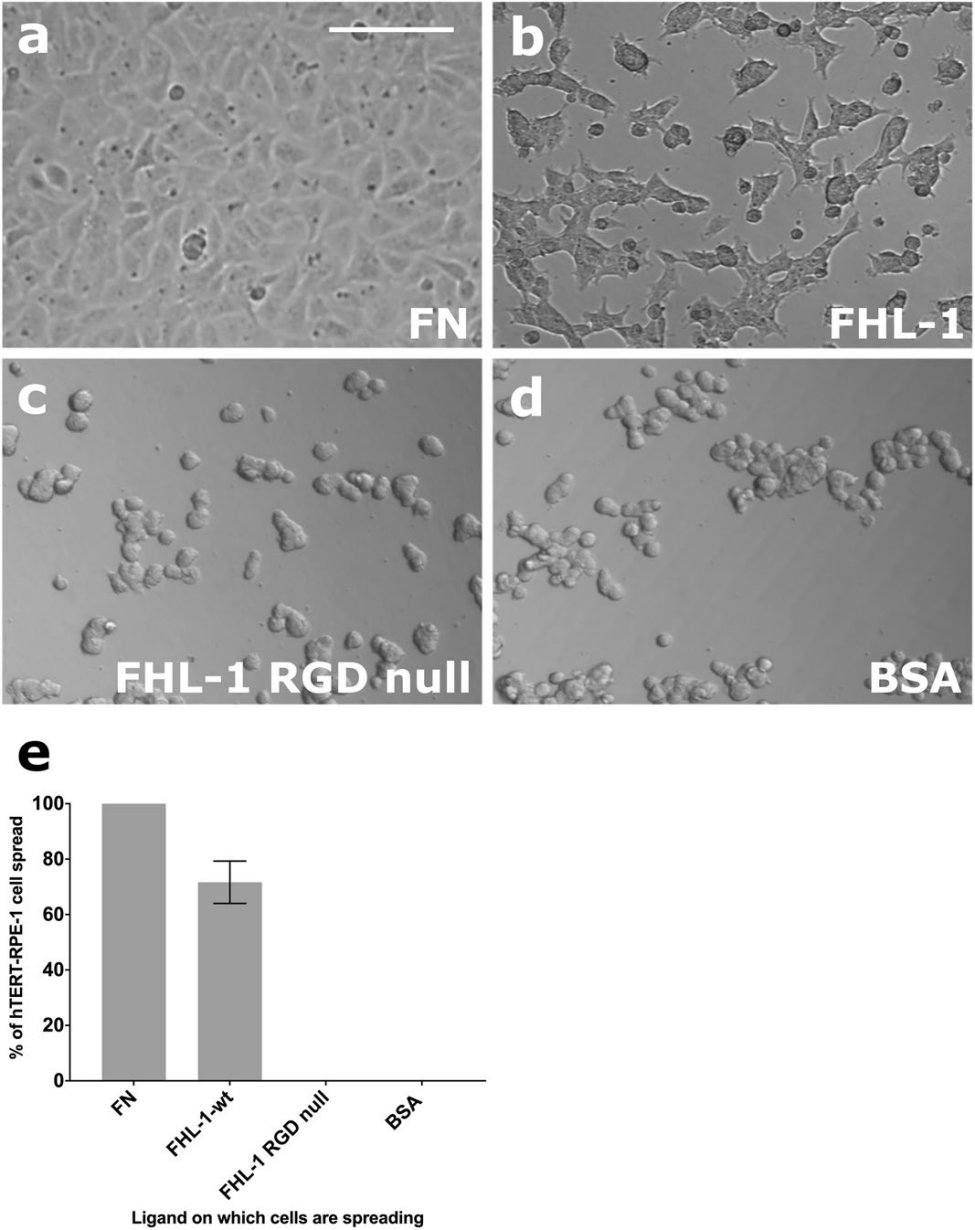
hTERT RPE-1 cells mirror the FHL-1 binding of primary RPE cells. The hTERT RPE-1 cell line was tested for FHL-1 binding by addition to different matrices at the same density (10,000 cells in each well of a 96-well plate), including (**a**) FN; (**b**) FHL-1; (**c**) FHL-1 RGD null; and (**d**) BSA. (**e**) the number of spread cells were counted in four different visual fields for each condition and each condition were measured in triplicate. The percentage of spread cells was calculated and compared to the positive control, FN. Data are the mean results of three independent experiments and each performed in triplicate. Images in (**a**-**d**) are representative of 3 independent experiments. Incubation was done for 3 h at 37 °C. Data in (**e**) represent n = 3 ± s.e.m. Scale bar represents 100 μm.

Next, we investigated the effects on gene transcription of the integrin/FHL-1 interaction, but surmised that this would be challenging using primary cells due to the naturally occurring donor-to-donor variability. Therefore, we tested the suitability of the RPE cell line hTERT RPE-1 for use in further experiments. Cell spreading assays were repeated as before and approximately 70% cell spreading was demonstrated on immobilised FHL-1 with hTERT RPE-1 cells when compared to FN (Fig. 5). No cell spreading was observed on the FHL-1 RGD null mutant control, confirming that the interaction was RGD-binding integrin mediated.

### Substrate-dependent alteration of gene expression in hTERT RPE-1 cells

In order to investigate the effects of different substrates on gene expression RNA-seq transcriptome analysis was carried out on hTERT RPE-1 cells grown for 24 h on three different substrates; FHL-1, FN and LA. Data were analysed using Ingenuity Pathway Analysis (IPA; Qiagen) software comparing differences between substrate in a core analysis with a false discovery rate (FDR) of 0.1. A further analysis was carried out using DAVID (https://david.ncifcrf.gov/): see Supplementary Tables 1–3.

For functional annotation clustering in DAVID analysis, gene lists were constructed comparing differential gene expression in cells between the different substrates: FHL-1 vs FN (201 differentially expressed genes), FHL-1 vs LA (139 genes) and LA vs FN (162 genes), see Supplementary Tables 1–3. Functional annotation clustering, Supplementary Tables 4–6, revealed that the majority of changes when comparing FHL-1 and FN concerned cell cycle and DNA replication, while in the case of comparing FHL-1 and LA, the highest enrichment occurred in the immune response and cytokine activity.

**Figure 6:**
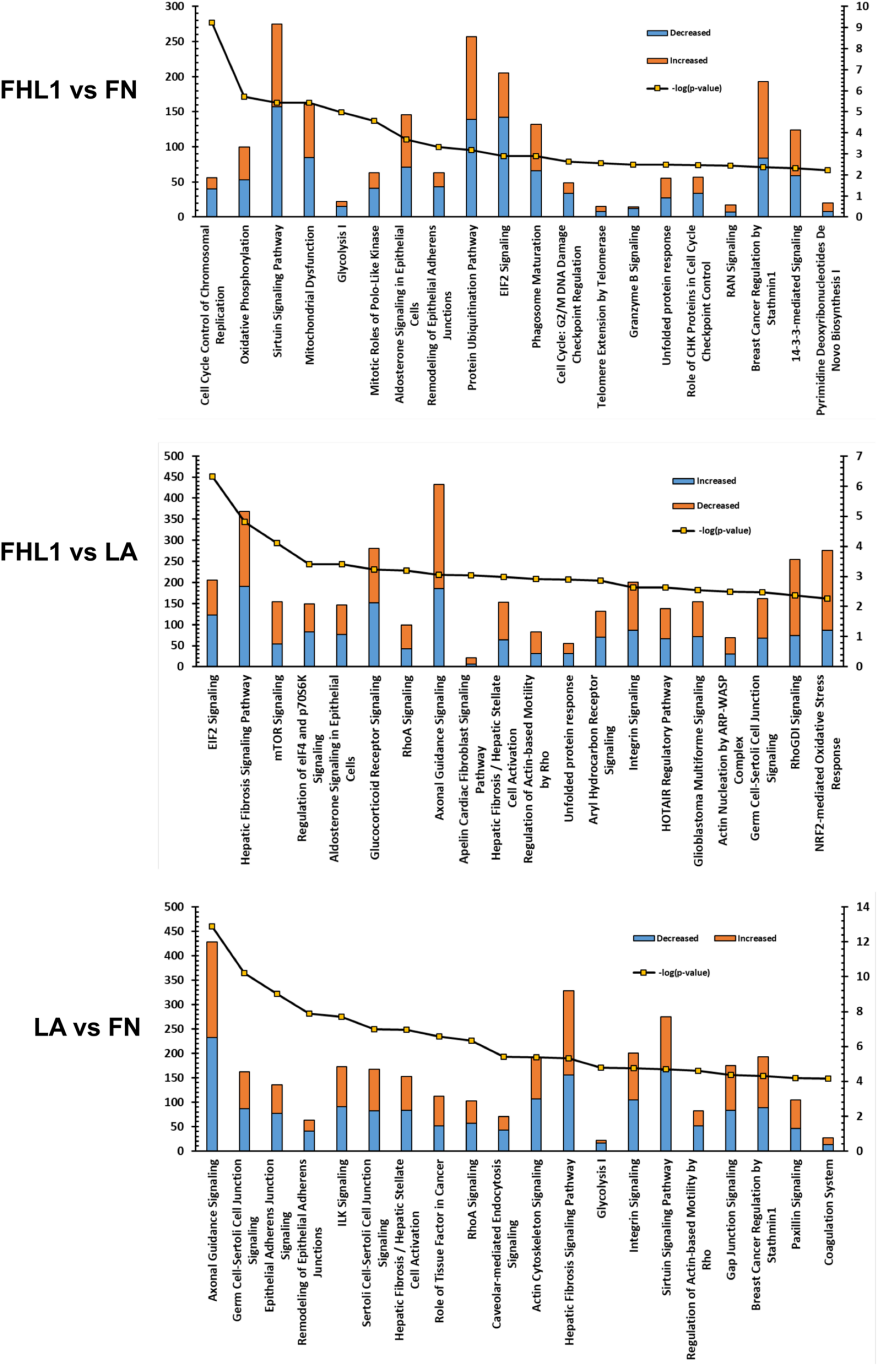
IPA analysis of differentially expressed genes in the most significantly altered canonical pathways. Bar charts indicate the most significantly different canonical pathways and the number of differentially expressed genes for each substrate comparison. Blue indicates decreased expression; orange genes indicate increased expression according to each comparison (measured on the left axis) and the line indicates significance as -log p value (measured on the right axis). “Data were analysed through the use of IPA (QIAGEN Inc., https://www.qiagenbioinformatics.com/products/ingenuitypathway-analysis).

IPA analysis also revealed similar differences in canonical pathways predicted to be altered when comparing between substrates (Fig. 6: all significantly altered pathways of interest are found in Supplementary Tables 7–9). In the case of FHL-1 vs FN, the majority of genes in the top 20 predicted pathways were found to be downregulated by FHL-1 especially those involved in the cell cycle and/or DNA repair, e.g. cell cycle control of chromosomal replication, mitotic roles of polo-like kinase, cell cycle G2/M DNA damage checkpoint regulation, telomere extension by telomerase, and role of CHK proteins in cell cycle checkpoint control. When comparing FHL-1 vs LA, the majority of genes were upregulated by FHL-1 in predicted pathways, these included cell/cell interaction pathways such as axonal guidance, and integrin signalling. There were no differences observed in integrin signalling when comparing FHL-1 vs FN, suggesting these two substrates regulate similar genes in that pathway. Additional pathways predicted to be altered in FHL-1 vs LA included intracellular cell signalling pathways, e.g. EIF2 signalling, mTOR signalling, nuclear receptor signalling pathways (glucocorticoid and aldosterone), Rho signalling pathways (RhoA, RhoGDI and actin-based motility by Rho). The NRF2-mediated oxidative stress response was also altered, suggesting that FHL-1 may exert anti-oxidant effects. A number of these pathways were commonly regulated in FHL-1 vs FN and FHL-1 vs LA but not in LA vs FN, and therefore are likely to be specific to FHL-1, i.e. unfolded protein response, aldosterone signalling in epithelial cells, and EIF2 signalling. Taking these data together, FHL-1 predominantly inhibits expression of genes controlling the cell cycle compared to FN and exhibits differences in cell/cell interaction mechanisms when compared to the effects of LA.

### FHL-1 protects hTERT RPE-1 cells from oxidative stress induced cell death

**Figure 7:**
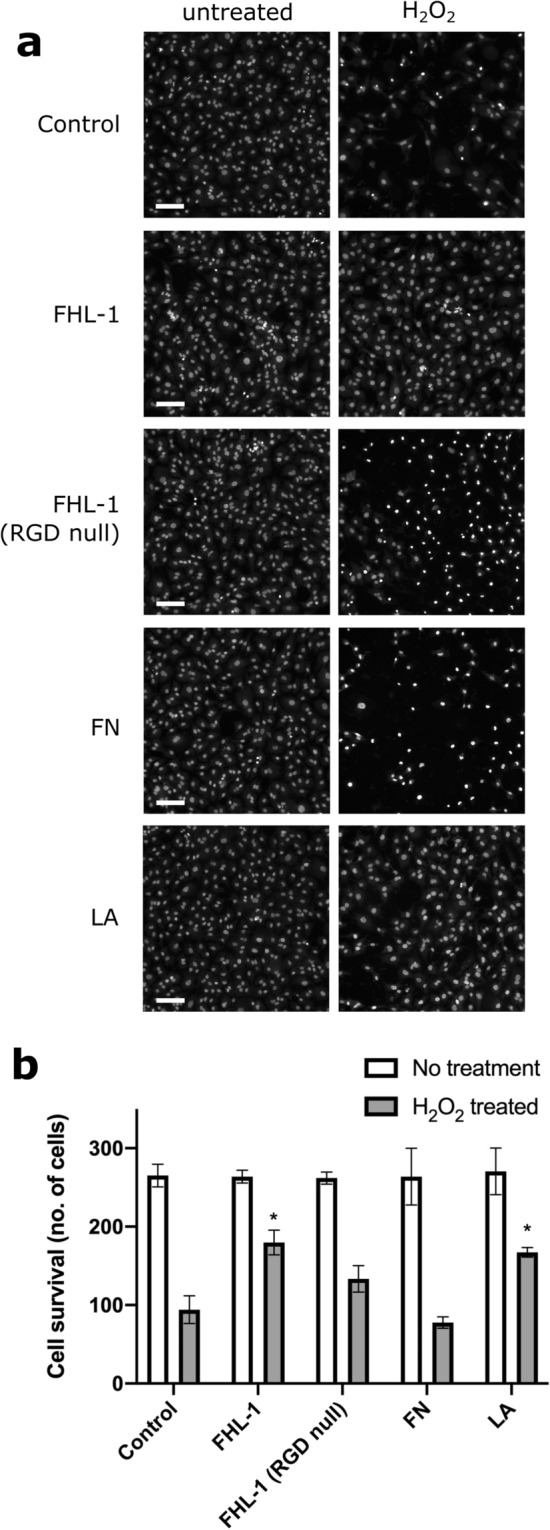
FHL-1 and LA protect hTERT RPE-1 cells from oxidative-stress induced cell death. (**a**) UV Photographs of cells grown on FHL-1, FHL-1 RGD null, LA and FN coated plates subsequently stained with Hoechst 33,342 after 24 h in the presence or absence of H_2_O_2_. Scale bar equals 100 μm. (**b**) Bar chart quantifying this data demonstrates that FHL-1 and LA exert significant protective properties compared to FN and a partial, but non-significant protective effect with FHL-1 NULL (paired One-Way ANOVA with Tukey post hoc analysis, where *P < 0.05). Experiment was done with cells grown for 24 h on ligands and then treated with H_2_O_2_ for further 24 h before staining. Experiment was repeated three times and in triplicate. Images in (**a**) are representative of three independent experiments.

As FHL-1 modifies oxidative stress gene expression, and the unfolded protein response via heat shock protein gene expression (including the *HSPA6* gene), we investigated the putative protective response of FHL-1 to oxidative stress-induced cell death. To this end, cell survival assays were performed on hTERT RPE-1 cells grown in wells pre-coated with FHL-1, RGD-null, FN, LA and PBS control for 24 h at concentrations similar to those used for the cell spreading assays. After switching to serum-free medium supplemented with B27 without antioxidants, cells were maintained with H_2_O_2_ (150 µM, a concentration we have found to kill approx. 50% of RPE cells in a variety of cell lines^32^) for a further 24 h. After this, cells were stained with Hoechst 33,342 and imaged using an automated scanner and counted using ImageJ. H_2_O_2_ treatment resulted in robust cell death in control cells (PBS coated wells) compared to untreated cells (Fig. 7). The number of cells in untreated wells were not significantly different when comparing different substrates indicating no differential effects on baseline survival. However, cells grown on FHL-1 and LA were significantly protected (p < 0.01 and p < 0.02 respectively) when compared to H_2_O_2_-mediated cell death on control substrate while cells grown on FN exhibited no such protection. FHL-1 RGD-null did not show a significant protective effect. These data identify a role of FHL-1 in mediating RPE cell survival against oxidative stress similar to that conferred by LA binding.

## Discussion

Here, we describe a novel interaction between human RPE cells and a protein associated with Bruch’s membrane, the complement regulator FHL-1. Despite the previously known important complement regulatory functions of FHL-1^7,18^, the interaction described in this study demonstrates a non-canonical role of FHL-1 in protecting RPE cells from oxidative-stress induced cell death. The human retina is one of the most metabolically active sites within the human body and the RPE cells are particularly subject to extensive levels of oxidative stress. There is strong evidence implicating oxidative stress in the pathogenesis of AMD^33–35^, and the stability of RPE cells in vivo is dependent on their interactions, not only with one another, but also their underlying ECM, Bruch’s membrane. Our short-term cell-spreading assays at three hours revealed an immediate α5β1-FHL1 protein–protein interaction while the separate RNAseq analysis after 24 h reveals longer term cellular mechanisms in RPE cells growing on FHL1, however these are not necessarily driven by the α5β1 integrin alone. The two sets of experiments address different questions and cannot be used to support nor dismiss the data obtained from each other. It is possible that other binding mechanisms supported by the presence of a mature ECM deposited by the RPE cells will also play a role in governing RPE cell gene expression and cell morphology. The observation that a lower percentage of cells attached to the FHL-1 substrate as compared to those attached to FN implies that there may be an underlying role for cell proliferation and/or cell death processes in the spreading assay. It has been reported that primary cells grown on FN show significant attachment in spreading assays and show an increase in cell number^36^. Indeed, our RNA-Seq studies and cell-survival studies in hTERT RPE-1 cells suggest that, in the case of FHL-1, there may be subtle decreases in expression of cell-cycle genes and no differences in proliferation. This however may be due to the effects of contact inhibition due to higher confluency. Therefore, experiments must be carried out in future with primary cells to examine the role of cell-death and proliferation in FHL-1-mediated spreading and attachment. Nevertheless, in our study reported here the majority of cells present did attach to FHL-1 via a mechanism involving interaction with α5β1.

RPE cell attachment to ECM components through integrins is a well-documented phenomenon^13,14^, and increasing RPE cell integrin expression has been postulated as a method for improving the adhesion of transplanted RPE cells to a recipient’s Bruch’s membrane^37^. Furthermore, integrin-mediated RPE adhesion to Bruch’s membrane confers protective effects^38,39^. Previous work investigating these interactions has focused on the main ligands of integrin receptors within Bruch’s membrane; collagen, LA, and FN^6^. By using cultured primary RPE cells, isolated from human donor eyes, we demonstrate their ability to interact with immobilised FHL-1 (see Fig. 2). Crucially, the cellular expression of the α5β1 integrin has been shown to be present in RPE cells in different states of differentiation and is expressed basally by human RPE cells in vivo^40^. Given that FHL-1 is identical to the first seven domains of FH (plus a unique four amino acid C-terminal tail: see Fig. 1b), and both have exactly the same RGD-containing CCP4^16,17^, it was of interest that RPE cells spread on immobilised FHL-1 but not FH. This phenomenon has been observed previously where a series of anchorage-dependent cell lines were shown to adhere to FHL-1, but not FH^27^. This study, which used recombinant fragments of the FHL-1 protein, identified the RGD binding domain of FHL-1 as being essential for these interactions. Furthermore, without direct testing the authors hypothesised that integrins were responsible given the reliance on bivalent metal ions for the interactions to be successful. Interestingly, they also observed ~ 50% cell spreading on FHL-1 when compared to FN, where in our study we observed ~ 40% with cultured primary RPE cells and ~ 70% with the hTERT RPE-1 cell line (Figs. 2 and 5). However, our subsequent competition of RPE cell/FHL-1 interactions with FH in the fluid phase (Supplementary Fig. 2) suggests that the lack of RPE cell interaction with immobilised FH is a result of how the protein adheres to plastic in our experimental settings rather than necessarily a representation of its lack of RPE cell adhesion in vivo. The predominance of FHL-1, rather than FH, within Bruch’s membrane^18^ may indicate that the RPE/ FHL-1 interaction is more important for oxidative stress induced gene expression, although this does not exclude a lesser involvement of FH in RPE cell resilience to oxidative stress, particularly in its fluid phase form.

Evidence of complement over-activation in the ECM of the choriocapillaris in AMD, and indeed preceding clinical manifestation of the disease, suggests complement gene variants that confer AMD-risk cause this dysregulation^41–45^. In addition, focus has recently been given to the non-canonical roles of complement proteins in AMD pathogenesis. For example, the common high-risk Y402H polymorphism in FH has been associated with lipoprotein dysregulation and causing an ocular phenotype in aged mice^26^. Also, the Y402H polymorphism hinders the ability of FHL-1 to bind glycosaminoglycans (GAGs), such as heparan sulphate^18,46–48^, although the presence of a secondary GAG-binding site in the full-length protein means FH itself is not affected^49^. The age-associated reduction in heparan sulphate chains in Bruch’s membrane^50^, coupled with the Y402H predisposition to weaker binding, means less FHL-1 will be present in Bruch’s membrane to regulate local C3b deposition and complement activation. This study suggests that the decrease in ECM-bound FHL-1 would also result in less RPE cell interactions with FHL-1 and consequently increase their susceptibility to oxidative stress.

We investigated the effects of different immobilised integrin ligands on hTERT RPE-1 cell gene transcription using FHL-1, FN and LA substrates. FN binds to similar integrins as FHL-1 (*ITGA5:ITGB1*) while LA does not. Considering the model used here for RNA-Seq was an immortalized cell line, a similar but not identical model to primary cells for which the original observations were made, analysis on the pathway level is much more appropriate for investigating putative changes, as pathways/gene networks tend to be regulated in a similar way, when using similar but not identical models while specific gene expression patterns can be variable^51^. RNAseq, Ingenuity Pathway Analysis, as well as functional annotation clustering using DAVID, uncovered altered activity in pathways that were specific to FHL-1 when compared to either FN or LA.

Compared to FN (which was not protective in the cell survival assays), cells grown on FHL-1 exhibited changes in genes associated with the cell cycle, including: Cell Cycle Control of Chromosomal Replication, Mitotic Roles of Polo-Like Kinase, Cell Cycle: G2/M DNA Damage Checkpoint Regulation and Role of CHK Proteins in Cell Cycle Checkpoint Control (see Supplementary Table **7**). The majority of genes in these pathways were down-regulated, but any putative effect on cell cycle by FHL-1 is likely to be subtle, as there were no differences in cell numbers when comparing hTERT RPE-1 cells on FHL-1 and FN in the cell survival assays (when not treated with hydrogen peroxide) (Fig. 7). Although there have been no previous studies on the role of FHL-1 on cell cycle control, mice deficient of full-length FH (also not expressing FHL-1) exhibit changes in the number of cells in the developing retina^25^. These *Cfh*^-/-^ animals displayed reductions in the rate of mitosis during a critical period of retinal development directly after birth. It is interesting to note that this study also observed enlarged mitochondria, observations consistent with premature ATP decline, degeneration and/or senescence. Ingenuity Pathway Analysis also identified changes in metabolic pathways involved in ATP production such as oxidative phosphorylation and glycolysis as well as mitochondrial dysfunction and sirtuin signalling, which is responsible for aging/senescence processes within the cell^52^. In relation to AMD, mitochondrial dysfunction and DNA damage occurs in the disease-associated Y402H variant of FH^53^ and this is presumed to be due to the increase in formation radical oxygen species and subsequent oxidative stress. Of course, it should be noted that these observations in hTERT-RPE1 cells may not be identical to gene expression profile changes that may occur in RPE cells in vivo. Indeed, further experimental studies are required to understand better the exact effect conferred by FHL-1 engagement by primary RPE cells in the human eye where the cells are present in a fully confluent cell monolayer.

Compared to hTERT RPE-1 cells grown on LA, the cells grown on FHL-1 exhibited altered expression in a number of pathways that were different to those observed when comparisons were made to FN. Interestingly, integrin signalling was one such pathway, and a component of that pathway, ITGA5 was found to be a significantly differentially expressed. FHL-1 and FN both bind to ITGA5:ITGB1. Therefore, it was interesting to observe when comparing gene expression changes in cells grown in LA with those grown on FN, the integrin pathway signalling was found to be an affected pathway, highlighting that FHL-1 and FN share common mechanisms in regulating integrin signalling. FHL-1 vs LA-specific changes included mTOR Signalling, a pathway important in maintaining retinal homeostasis in RPE cells by regulating lysosomal phagocytosis and autophagy^54^, and NRF2-mediated Oxidative Stress Response that may confer protection to oxidative insults^55^. Studies have previously linked abnormal autophagy with reduced NRF2 signalling in animal models of AMD^56^.

HSPA6, which is an inducible form of HSP70, was found to be the highest upregulated gene when cells were grown on immobilised FHL-1 when compared to either FN or LA. HSP6A which is only partially conserved in the mammalian lineage and has no homologs in rodents has been reported to be induced in an in vitro model of photocoagulation in the ARPE-19 cell line^57^. HSPA6 and another inducible HSP70 gene, HSP1A, which was also demonstrated to have increased expression in cells grown on FHL-1, work in tandem to protect cells against proteotoxic insults and heat shock-mediated cell death in various cell lines^58,59^. HSP70 proteins are also known to interact with mineralocorticoid receptor (MR), a high affinity ligand of aldosterone, and keep it in a basal state^60^. In this non-activated state, MR is predominantly cytoplasmic and part of a large heteromeric complex interacting with a number of proteins including HSPs. Upon ligand binding, a conformational change occurs that leads to the dissociation of the complex and subsequently the MR translocates to the nucleus binding to DNA leading to the regulation of gene transcription. Aldosterone binding and activation of MR signalling pathways are associated with increased levels in oxidative stress in vascular inflammation^61^ and this is relevant to certain retinal disorders. Use of the MR antagonist spironolactone, reduces CNV activity in patients with refractory neovascular AMD in a VEGF-independent manner^62^. Therefore, an increase in the level of heat shock proteins (and thus increased sequestration of MR to the cytoplasm) is one potential mechanism by which FHL-1 may illicit a protective response to RPE cells from oxidative insult.

FHL-1-specific cytoprotective effects may be mediated via the unfolded protein response, a pathway found to be altered in both FHL-1 v FN and FHL-1 v LA comparisons. This pathway which includes HSPs, restores normal cell function by halting transcription in response to misfolded proteins and ER stress^63^. This process occurs by activating degradation of misfolded proteins which would otherwise enter the mitochondria and cause dysfunction in energy production. Downstream degradation pathways including ER-associated degradation and ubiquitin–proteasome system, endo-lysosomal and autophagy may be putatively affected^64^ although only the protein ubiquitination pathway is a top altered pathway when comparing FHL-1 vs FN.

These findings point to a putative role of FHL-1 in mediating a response to oxidative stress. Therefore, we tested the effect conferred by FHL-1 with RPE cell survival in a hydrogen peroxide-induced oxidative stress model. While immobilised FHL-1 exhibited protective effects similar to that observed with immobilised LA, cells grown on FN did not survive to the same extent (Fig. 7). This indicates that any mechanism of FHL-1 mediated protection occurs independently of any interaction with the integrin signalling pathway shared with FN. The protective effect is not due to changes in cell cycle, as the number of cells that grew on immobilised FHL-1 (and untreated with hydrogen peroxide) were similar to those of untreated cells grown directly on plastic. No previous studies have demonstrated that FHL-1 can confer a protective effect against oxidative stress; however, a recent study has shown that full length FH, when supplemented to the cell culture medium can protect ARPE-19 and human iPSC-derived RPE cells from oxidative insult as a result of 4-HNE treatment^65^. Furthermore, Borras et al. found that FH inhibits caspase-induced apoptosis and protects RPE tight junctions from oxidative stress-induced disruption^65^. However, the Borras et al*.* study did not identify how the full-length protein was interacting with the RPE cells to confer these effects as recombinant truncated fragments of FH all failed to replicate the results seen with the full-length protein. Again, it would be pertinent to extend this study by performing further studies in an attempt to confirm these observed improvements in cell survival in RPE cells more physiologically akin to those found in vivo.

Another intriguing, although untested, implication for our data lies in a potential role of FHL-1/RPE cell integrin interactions in wound healing, particularly with regard to proliferative vitreoretinopathy (PVR), the most common cause of failure for rhegmatogenous retinal detachment repair^66^. PVR is characterized by the growth and contraction of cellular membranes within the vitreous cavity and on both sides of the retinal surface as well as intraretinal fibrosis^67^. In this situation, mature differentiated RPE can become detached from Bruch’s membrane and lose their usual polarity and entering EMT, a state far more comparable with that of the immature RPE cells seen in cell spreading assays, than mature polarized RPE. Cell surface receptor integrins regulate a range of physiological functions as well as sense ECM-induced extracellular changes during wound healing, leading to cellular responses that influence ECM remodelling^68^. Previous studies also showed that integrins like αVβ6 promote corneal wound healing^69^ and αVβ5 in dermal wound healing^70^. Given that blockage of the αVβ5 integrin in our study also inhibited RPE cell interactions with immobilised FHL-1 (Fig. 3) it remains possible that interactions between RPE cell integrins and FHL-1 in Bruch’s membrane could play an as of yet undefined role in RPE cell attachment and response to PVR. Furthermore, given the nature of geographic atrophy in dry AMD and the failing health of RPE cells around the edge of the associated lesion, a link to similar mechanisms underlying wound healing and PVR may be envisioned, although clearly any such hypothesis will require extensive testing in future studies.

Our study has been the first to suggest a role of FHL-1 in mediating RPE cell resistance to oxidative stress and highlights the need to fully understand how RPE cells interact with their underlying ECM. Here we suggest that RPE cell gene transcription may be modulated, directly or indirectly, by interaction of RPE α5β1 integrin receptors with FHL-1. Our work highlights a potential new avenue of scientific investigation that requires further work, especially testing this hypothesis in an in vivo setting. This adds a novel function to the repertoire of FHL-1 by mediating both a complement response as a co-factor for FI in this micro-environment, but also confers a protective effect against oxidative insults on the RPE cells. This novel finding will help in our understanding of RPE cell behaviour in vivo, and highlights the need to consider the endogenous RPE cell/ECM interactions when designing therapeutic interventions.

## Materials and methods

### Primary RPE isolation from human eye-globes

Human eyes were collected from the Manchester Royal Eye Hospital Eye Bank after removal of the corneas for transplantation. Informed consent had been obtained for the eye tissue to be used for research and guidelines established in the Human Tissue Act of 2004 (UK) and the tenets of the Declaration of Helsinki were adhered to. Ethical approval for the use of human donor eyes was given by North West – Greater Manchester Central Research Ethics Committee (REC reference 15/NW/0932). A total of 22 donor eye pairs were used (13 female, 9 male) with an age-range between 42 and 77 years old (mean age 58.8 years old). All donors used were < 48 h post mortem. Eye globes were collected in collection media (Hank’s Balanced Salt Solution (Sigma-Aldrich, Poole, UK) supplemented with 1% (w/v) Amphotericin-B, 1% (w/v) Penicillin/Streptomycin, 0.5% (w/v) Sodium Pyruvate, 1% (w/v) HEPES, 0.01 mg/ml gentamycin (Sigma-Aldrich). Each eye globe was placed in a 10 cm petri dish and washed with phosphate-buffered saline (PBS). Then the iris, lens, vitreous and neurosensory retina (NSR) were gently removed from the posterior eye-cup using a scalpel, forceps and scissors. After that, the eye cup was rinsed with sterile PBS and the interior was digested using 0.5 mg/ml each of collagenase Type I-A (from *Clostridium histolyticum*, Sigma-Aldrich) and Type IV (from *Clostridium histolyticum*, Sigma-Aldrich) in 5 ml of sterile-filtered Dulbecco’s Modified Eagle’s Medium (DMEM, Sigma-Aldrich) media with high glucose for 90 min at 37 °C. Next the media containing the collagenases in the eye cup was gently discarded, then the eye cup was filled with 5 ml of fresh DMEM media and the RPE were gently scraped off the underlying Bruch’s membrane.

The media containing RPE sheets/fragments was collected and centrifuged at 150* g* for 5 min. The supernatant was removed and the RPE sheets/fragments were resuspended in 4 ml RPE media (1:1, DMEM: Ham’s F12 medium (GIBCO 31,330–038), 10% (v/v) foetal calf serum, 1% (w/v) penicillin–streptomycin). Resuspended RPE was added to 0.5% (w/v) gelatin (Sigma-Aldrich)-PBS coated-wells of 12 or 24 well plates. After 24 h, the RPE was washed 3 × with PBS and fresh media was added. RPE cells were grown until they were confluent and ready to be passaged.

### Clonal cell line tissue culture

The human immortalized RPE cell line hTERT RPE-1 (ATCC CRL-400) was purchased from ATCC (LGC Standards, UK). The hTERT RPE-1 cells were grown in DMEM: Ham’s F-12 (ATCC, 1:1) media containing 10% (v/v) foetal calf serum (ATCC), 100 U/ml penicillin and 100 mg/ml streptomycin and 0.01 mg/ml hygromycin B (Sigma-Aldrich). HEK293T cells were grown in DMEM, containing 10% (v/v) fetal calf serum, 100 U/ml penicillin and 100 mg/ml streptomycin. All cells were maintained at 37 °C in 5% humidified CO_2_.

### Transfection of wild-type and RGD-null FHL-1 plasmid into HEK293T

cDNA encoding histidine-tagged full-length wild-type FHL-1^18^ and the RGD-null FHL-1 protein, where the aspartic acid is substituted with an alanine to disrupt the RGD binding site was synthesised commercially (Life Technologies, Paisley, UK). Purified cDNA was incorporated into a pcDNA3.1 vector and transfected into HEK293T cells using PEI max transfection reagent (Polysciences, Germany) as described previously^18^. Briefly, for each 15 cm dish, 1 mg/ml plasmid DNA and 7.5 mM PEI were added to separate aliquots of 150 mM NaCl and each incubated for 10 min at room temperature. After incubation, the PEI solution was slowly added to the solution containing DNA and then incubated for 10 min at room temperature. The DNA/PEI mixture was added to a 15 cm diameter dish containing 7 × 10^6^ HEK293T cells/per dish in a dropwise manner and incubated at 37 °C. After 5–6 h, the media was replaced with 2% (v/v) FCS containing DMEM (no antibiotic) and incubated at 37 °C for overnight. Conditioned media was collected daily and replaced with fresh media over the next 4 days.

### Purification of wild-type and RGD-null FHL-1

Conditioned media from each of the 20 dishes (17.5 ml/dish) were collected and pooled after 24, 48, 72, and 144 h. Total conditioned media after 144 h (1,400 mls total) was diluted with the addition of 600 mls 50 mM HEPES, 500 mM NaCl, 20 mM Imidazole, pH 7.5. To this, 12 ml of NiNTA resin (Expedeon) was added and incubated overnight with rotation at 4 °C. The NiNTA beads were collected by passing the media through empty PD10 columns with a filter (GE Healthcare) by gravity flow (500 ml media per PD10 column, i.e. four PD10 columns in total). The beads were then washed with 10 ml of wash buffer (50 mM HEPES, 500 mM NaCl, 20 mM Imidazole, pH 7.5). Finally, wild-type FHL-1 or RGD-null FHL-1 protein was eluted using 16 ml of elution buffer (50 mM HEPES, 500 mM NaCl, 500 mM imidazole, pH 7.5). Eluted protein was dialysed back into wash buffer overnight at 4 °C before being concentrated further by addition to 1.5 ml NiNTA beads and eluted in 6 × 1 ml aliquots. Purified protein aliquots were dialysed into 20 mM glycine, 125 mM NaCl, pH 9.0 using a dialysis cassette Slide-A-Lyzer (Fisher, cat no 10759784) with a 10 kDa cut off. The purified recombinant proteins (i.e. FHL-1 and RGD-null FHL-1) were assessed for purity by SDS-PAGE, and visualised by staining the gels for 60 min at room temperature with Instant Blue Coomassie stain (Expedeon, Cambridge, UK).

### Endotoxin testing of purified FHL-1 proteins

The endotoxin level of FHL-1 recombinant protein preparations was measured using the Toxin Sensor Chromogenic LAL Endotoxin Assay Kit (GenScript, NJ, USA), according to the manufacturer’s protocol. This method utilizes a modified Limulus Amebocyte Lysate (LAL) and a synthetic colour producing substrate to detect endotoxin chromogenically. Briefly, 100 µl of standards (0, 0.01, 0.025, 0.05, 0.1 and 0.5 EU/ml), test samples (recombinant wild-type FHL-1 and RGD-null FHL-1 proteins) and a blank containing 100 μl of LAL reagent water were dispensed into endotoxin-free vials in duplicate. 100 µl of reconstituted LAL was added to each vial, mixed by swirling and incubated at 37 °C in a water bath. After incubation, 100 µl of reconstituted chromogenic substrate solution was added to each vial and mixed gently to avoid foaming. All the vials were further incubated at 37 °C for 6 min. Finally, 500 µl of each of three colour stabilisers were added eventually to each vial and mixed gently to stop the reaction. The absorbance of each reaction vial was read at 545 nm.

Under the standard conditions, the absorbance at 545 nm shows a linear relationship within a concentration in the range of 0.01 to 1 EU/ml. The absorbance for the five standards was plotted on the x-axis and the corresponding endotoxin concentration in EU/ml on the y-axis. A best-fit straight line was drawn between these points and the endotoxin concentrations of samples were determined graphically.

### Fluid-phase cofactor activity of wild-type and RGD-null FHL-1 (C3b break-down assay)

To test the functional capacity of both FHL-1 and RGD-null FHL-1, a C3b breakdown assay was employed as described previously^23^. Briefly, 0.1 µg of either wild-type FHL-1 or RGD-null FHL-1 was incubated with 2 µg C3b (VWR International, Lutterworth, UK), and 0.4 µg factor I (FI) (VWR International,) in PBS (total volume of 20 µl) for 15 min at 37 °C. The reaction was stopped by the addition of 5 µl 5 × SDS reducing sample buffer (NuPAGE LDS sample buffer, Life Technologies) and boiling for 10 min at 100 °C. Samples were run on a 4–12% NuPAGE Bis Tris gels (Life Technologies, UK) at 150 V for 75 min to allow the separation of the C3b breakdown product bands. Blue Protein Standard Broad Range (New England Biolabs, Hitchin, UK) was used as a protein marker. The gels were stained using Instant Blue for 1 h at room temperature. Gel images were taken using an Alpha Innotech FluorChem 5500 gel imaging system.

### Immunofluorescence staining

Primary RPE cells were seeded on wells of a 6-well plate with 4 coverslips in each well and grown for 24 h at 37 °C. Cells were fixed in 4% (w/v) paraformaldehyde (PFA) for 20 min at 4 °C. PFA was removed and the coverslips were washed with PBS. Cells were permeabilised with 0.5% (v/v) Triton X-100 in PBS for 10 min at room temperature. After washing with PBS, the seeded coverslips were blocked with 5% (v/v) normal goat serum (NGS) for 1 h at room temperature. These were then washed three times with PBS and labelled with antibodies directed against RPE cell markers including mouse monoclonal anti-RPE65 (Abcam, clone: 401.8B11.3D9) and anti-bestrophin-1 (Novus Biologicals, UK, clone: IgG1 E6-6), or the tight-junction marker anti-ZO-1 (Invitrogen, clone: ZO-1-IA1Z). All antibodies were diluted 1:50 in PBS containing 5% (v/v) normal goat serum (NGS; 100 µl/coverslip) (Novus Biologicals, UK) and incubated overnight at 4 °C. Cells were washed with PBS and then incubated with secondary antibody Alexa Fluor 488-conjugated goat anti-mouse antibody (Life Technologies) diluted at 1:100 in 5% (v/v) NGS in PBS for 1 h at room temperature. Finally, DAPI was applied as a nuclear counter stain at 0.3 µM for 5 min prior to mounting with medium (Vectashield soft, Vector Labs, Peterborough, UK) and placing the coverslips upside down onto microscope slides. Images were taken using by a snapshot widefield microscope (Leica, 20 × /0.50 PL FLUVOTAR objective) using HCImage software.

### Cell spreading assay

For the cell spreading assays, 96-well plates were coated with different protein ligands (50 to 80 µg of FHL-1, FH (HyCult, Uden, The Netherlands), FHR-4^23^ and 10 µg of FN (Sigma-Aldrich, cat F1141) and BSA) in PBS (with Ca^2+^ and Mg^2+^, Sigma-Aldrich) and incubated overnight at 4 °C. Then non-specific binding was blocked by heat-denatured 1% BSA in PBS (heated for 11 min at 85 °C) for one hour at room temperature. Primary RPE or hTERT RPE-1 cells were trypsinized, counted using haemocytometer and 10,000 cells were added in each well for each condition in triplicate. Cells were then allowed to spread for 3 h at 37 °C in 5% CO_2_ incubator. For quantification of cell spreading, cells were photo-documented (Leica microscope, 10 × /0.22 HI PLAN objective and DFC420 camera) with at least four separate images being taken from four separate fields in each well. These images were analysed using Leica imaging software and ImageJ was used to count the cells. Cell spreading was characterized by a cytoplasmic halo around the cell nucleus. RPE cells showing this phenomenon were counted as ‘spread’ and with ‘round’ shape were counted as ‘non-spread’ cells. The percentage of the total number of cells per field was calculated.

### Inhibition and competition assay

96-well plates were coated with different protein ligands (80 µg FHL-1, FH, FHR-4 and 10 µg of FN and BSA) in PBS (with Ca^2+^ and Mg^2+^, Sigma-Aldrich) and incubated overnight at 4 °C. Then all coated wells were blocked with heat-denatured 1% (w/v) BSA in PBS for one hour at room temperature. Primary RPE and hTERT RPE-1 cells were trypsinized and counted using a haemocytometer. Cells (in each condition 10,000 cells/well in triplicate) were incubated with 10 µg/ml of mouse monoclonal anti-α5 (mAb16), anti-β1 (mAb 13), anti-αV (17e6), anti-αVβ3 (LM609) or anti-αVβ5 (PIF6) integrin antibodies (all kindly supplied by the Prof. Humphries lab), for 20 min at room temperature before adding to the wells and then incubated for 3 h at 37 °C to allow cells for spreading.

In other inhibition assays, cells were incubated with increasing concentrations (0 to 120 µg/ml) of a reverse sequence control peptide (Ser-Asp-Gly-Arg-Gly/SDGRG, Sigma-Aldrich) and an RGD peptide (Gly-Arg-Gly-Asp-Ser/GRGDS, Sigma-Aldrich) for 20 min at room temperature before the cells were added to the wells. Cells were then allowed to spread for 3 h at 37 °C.

In competition assays, either primary RPE or immortalized hTERT RPE-1 cells were pre-incubated with different proteins such as FHL-1 (50 µg/ml), CCP6-7 of FHL-1^71^ at 50 µg/ml, FH (150 µg/ml) and FN (10 µg/ml) for 20 min at room temperature before being added to FHL-1 (50 µg/ml) coated plates. To allow the spread, cells were then incubated for up to 3 h at 37 °C.

To measure the cell spreading, four separate images of cells were photographed from four separate fields in each condition. These images were analysed using Leica imaging software and ImageJ was used to count the spread cells. Cell spreading was displayed by a cytoplasmic halo around the cell nucleus. Rounded cells were counted as ‘non-spread’ cells. The percentage of the total number of cells per field was calculated. Both inhibition and competition assays were repeated three times and performed in triplicate.

### RNA isolations and quantitative RT-PCR (qPCR)

The hTERT RPE-1 cells (1 × 10^5^ cells per well) were grown on either 50 µg/ml FHL-1, 10 µg/ml FN or 10 µg/ml LA (Merck Millipore, cat. No. CC095) substrate in a 24-well plate for 24 h at 37 °C. For gene expression analysis, total RNA was extracted from the attached cells using an RNA Isolate II Mini Kit (BioLine) according to the manufacturer’s protocol. RNA quality and concentration were measured with a NanoDrop 2000 spectrophotometer (ThermoFisher Scientific). cDNA was synthesized using 1 µg of RNA and a High Capacity cDNA Reverse Transcription kit (Applied Biosystems) according to the manufacturer’s instructions. PCR was carried out using TaqMan Gene Expression Assays (FAM/MGB-NFQ, ThermoFisher Scientific, Paisley, UK) and real time PCR (qPCR StepOne Plus, ThermoFisher) cyclers. PCR reactions were carried out on 96-well plates (MicroAmp Fast Optical 96-well Reaction plate, Applied Biosystems, Life Technologies) and 2 µl cDNA (10–20 ng diluted in nuclease-free water) was added to each well in triplicate.

### Gene transcriptomic analysis by RNA-seq

For RNA-seq, hTRERT-RPE1 cells were grown to 80% confluency on either FHL1 (20 µg/ml), FN or LA (10 µg/ml). For each analysis 2 wells of a six-well plate were used and 3 replicates were performed (n = 9 in total). After media was fully aspirated from the wells, total RNA was isolated using an Isolate II RNA Mini Kit (Bioline) directly from the wells as per manufacturer’s instructions, and RNA quality and concentration measured using a Nanodrop 2000 spectrophotometer. Unmapped paired-end sequences from an Illumina HiSeq4000 sequencer were tested by FastQC (http://www.bioinformatics.babraham.ac.uk/projects/fastqc/). Sequence adapters were removed and reads were quality trimmed using Trimmomatic_0.36^72^. The reads were mapped against the reference human genome (hg38) and counts per gene were calculated using annotation from GENCODE 27 (http://www.gencodegenes.org/) using STAR_2.5.3^73^. Normalisation, Principal Component Analysis, and differential expression were calculated with DESeq2_1.16.1^74^. For differential transcriptional analysis, data were analysed through the use of IPA (QIAGEN Inc., https://www.qiagenbioinformatics.com/products/ingenuitypathway-analysis) comparing differences between substrates in a core analysis with a global false discovery rate (FDR) of 0.1. Analysis was also carried out using DAVID (https://david.ncifcrf.gov/) with a fold change cut-off of 1.5 when comparing between gene expression between substrates, transcripts with counts < 50 were defined as being absent.

### Cell survival assay

hTERT RPE-1 cells at 80% confluency were maintained in DMEM:F12 (1:1) media supplemented with 10% (v/v) FCS, 1% (w/v) penicillin–streptomycin and 0.01 mg/ml hygromycin B (all from Thermo Fisher Scientific UK). For experiments, cells were plated at a density of 100,000 cm^-2^ in 96 well plates (Corning, MERCK UK), with wells previously coated for 24 h with 20 µg/ml FHL-1, 20 µg/ml RGD null FHL-1, 10 µg/ml FN (Sigma-Aldrich), 10 µg/ml LA (Sigma-Aldrich) or 1 × PBS with added Ca^2+^ and Mg^2+^ (ThermoFisher Scientific UK) vehicle. After a further 24 h cells were switched to serum-free DMEM:F12 (1:1) medium supplemented with B27 without antioxidants (Thermo Fisher Scientific, UK). Cells from each group of coated wells were incubated in the above media in the presence or absence of 150 µM H_2_O_2_ (Sigma-Aldrich) for a further 24 h. Cells were then fixed in 4% paraformaldehyde (Sigma-Aldrich) in 1 × PBS for 15 min at RT and washed three times in PBS. During the second wash, 1 µg/ml Hoechst 33,342 (Sigma-Aldrich) added to the PBS wash to stain nuclei. Cells were imaged using a CellInsight CX5 scanner (Thermo Fisher Scientific) with a 10 × objective, taking three snapshots per well in identical positions, and three wells for each condition per experiment for three experiments. Cell counting was carried using the ImageJ software package. After thresholding to create a binary image, cell nuclei sized between 0–150 pixel^^2^ were included in counts. Numbers using these settings were consistent with manual counting from images from PBS control and PBS H_2_O_2_-stimulated cells.

### Statistical analysis

In competition assays whereby anti-integrin antibodies (anti-α5, anti-β1, anti-αV, anti-αVβ3, or anti-αVβ5) or purified proteins (FH, FHL-1, CCP6-8, FN) were used to disrupt RPE cell spreading on different matrices, images were taken from three separate fields of view for each condition per well and three wells for each condition in any one experiments. Finally, the experiment itself was repeated three independent times. Paired One-Way ANOVA non-parametric analysis with Tukey post hoc analysis was performed on the data to identify which anti-integrin antibody treatments that significantly reduced the level of cell spreading compared to the non-treated control, where *P* < 0.05 was considered significant.

Similarly, for the cell survival assays, Hoechst staining of cell nuclei were counted taking three snapshots per well in identical positions, and three wells for each condition per experiment for three separate experiments. Again, paired One-Way ANOVA non-parametric analysis with Tukey post hoc analysis was performed to identify which cell-spreading matrix made a significant improvement in cell survival compared to control (RPE cells grown directly onto plastic). In all cases, statistical analyses were performed using GraphPad Prism 9 software.

## Supplementary Information

Supplementary Information 1.

Supplementary Information 2.
